# Sibe: a computation tool to apply protein sequence statistics to predict folding and design in silico

**DOI:** 10.1186/s12859-019-2984-1

**Published:** 2019-09-06

**Authors:** Ngaam J. Cheung, Wookyung Yu

**Affiliations:** 1 Department of Brain and Cognitive Science, DGIST, Daegu, 42988 South Korea; 2 Cavendish Laboratory, Department of Physics, University of Cambridge, Cambridge, CB3 0HA UK; 3 Core Protein Resources Center, DGIST, Daegu, 42988 South Korea

**Keywords:** Evolutionary coupling analysis, Protein folding, Computational protein design, Protein structure prediction

## Abstract

**Background:**

Evolutionary information contained in the amino acid sequences of proteins specifies the biological function and fold, but exactly what information contained in the protein sequence drives both of these processes? Considerable progress has been made to answer this fundamental question, but it remains challenging to explore the potential space of cooperative interactions between amino acids. Statistical analysis plays a significant role in studying such interactions and its use has expanded in recent years to studies ranging from coevolution-guided rational protein design to protein folding in silico.

**Results:**

Here we describe a computational tool named Sibe for use in studies of protein sequence, folding, and design using evolutionary coupling between amino acids as a driving factor. In this study, Sibe is used to identify positionally conserved couplings between pairwise amino acids and aid rational protein design. In this process, pairwise couplings are filtered according to the relative entropy computed from the positional conservations and grouped into several ’blocks’, which could contribute to driving protein folding and design. A human *β*_2_-adrenergic receptor (*β*_2_AR) was used to demonstrate that those ’blocks’ contribute the rational design for specifying functional residues. Sibe also provides folding modules based on both the positionally conserved couplings and well-established statistical potentials for simulating protein folding in silico and predicting tertiary structure. Our results show that statistically inferences of basic evolutionary principles, such as conservations and coupled-mutations, can be used to rapidly design a diverse set of proteins and study protein folding.

**Conclusions:**

The developed software Sibe provides a computational tool for systematical analysis from protein primary to its tertiary structure using the evolutionary couplings as a driving factor. Sibe, written in C++, accounts for compatibility with the ’big data’ era in biological science, and it primarily focuses on protein sequence analysis, but it is also applicable to extend to other modeling and predictions of experimental measurements

**Electronic supplementary material:**

The online version of this article (10.1186/s12859-019-2984-1) contains supplementary material, which is available to authorized users.

## Background

A protein’s biological function and structure are evolved properties and are stabilized by thousands of weak interactions [1]. That interaction network is the foundation of how a protein works and is critical to understanding a protein’s origin via evolutionary processes and important for engineering new proteins and drugs. One such evolutionary process is coevolution, which refers to the coordinated changes that occur in pairs of biomolecules or residues to maintain or refine functional interactions between those interacting partners. Coevolution is a classic topic in biology for understanding the relationships between biomolecules or residues [2]. Although numerous coevolution-inspired computational methods have been developed for inferring these interaction networks, full descriptions of network coevolution that can fully describe functional and physical relationships remains challenging.

To better understand what coevolutionary information encoded in protein sequences is necessary and sufficient for protein folding or design, computational and statistical approaches have been applied to study effects of mutations, especially co-variations that may result in altered protein function and conformational changes [3–10]. Two striking representatives of such approaches are statistical coupling analysis (SCA) and direct couplings analysis (DCA). SCA measures the conservation-weighted correlation of positions in aligned homologous sequences of a protein family and detects physically connected networks of amino acids that link the main functional site to distantly positioned allosteric sites [4, 11, 12]. DCA [7, 9, 13] is an approach to predict direct tertiary contacts in protein structures using the top couplings [14].

Protein design has been a long-standing challenge to test computational approaches used in protein sequence analysis, folding, and structure prediction [15–21], Also referred to as an inverse folding problem, protein design seeks to create idealized proteins composed of canonical structural elements [22], including the design of coiled coils, repeat proteins, TIM barrels, and Rossman folds [15–20]. Consequently, statistical approaches aim to bridge the gap between protein sequence and design, which may be achieved if the approaches can predict protein stability and foldability. However, it is expensive and challenging to design functional assays to experimentally test such statistical approaches [23], although a designed WW-domain proteins have been created based on the SCA method and experimental demonstration [12]. Moreover, until recently, although SCA-based and DCA-based approaches have been applied to protein design, most of the statistical methods have focused on evolutionary sequence conservation analysis and predictions of residue-residue contacts [24], which usually are used as constraints for protein structure prediction [13, 25].

Our ability to reliably detect coevolutionary information will benefit from the development of additional systematic and well-packed tools that can efficiently and rapidly extract evolutionary information from protein sequences. Accordingly, we developed an end-to-end platform, termed Sibe, to investigate how positionally conserved couplings inferred from sufficiently large and diverse multiple sequence alignments (MSAs) can be used for specifying a protein’s structure and function and to build an improved version of the protein. As a general framework, Sibe aims to reduce the gap between sequence analysis and protein folding and design. Sibe provides an easy and rapid method for protein design and folding in silico using analytical and computational inferences based on protein sequences and estimated ‘residue blocks’ identified from highly correlated coevolution.

Sibe utilizes a combination of mathematical principles underlying SCA- and DCA-based methods for detecting patterns of structural contacts and functional couplings within sequence alignments to identify functional and physical interactions between amino acids [26]. In addition, positionally conserved couplings estimated by evolutionary coupling analysis (ECA) from a protein MSA to define rules for in silico prediction of the folding pathways and tertiary structure of a given sequence.

In this report, we provide two instructive examples to show the capabilities of Sibe for protein sequence analysis, protein design, and folding predictions. In the first example, we use Sibe to statistically analyze an MSA of a eukaryotic signal transduction protein, a G-protein coupled receptor (GPCR) [27], for detection of ’residue blocks’ and design in silico. We then use Sibe to build a mutated GPCR protein based on inferred coevolutionary information and compare the functional residues identified to those in ref. [27]. In the second example, Sibe is used to simulate the folding of a group of three proteins based on statistical potentials [28] and positionally conserved couplings.

## Implementation

The general procedure for launching Sibe is to define a set of protein sequences and then align them in order to estimate variation frequencies in the sequence alignment. Before the initial statistical analysis, we must obtain the sequences of a given protein we are interested in, and then analyze the multiple sequence alignment for capturing the coevolution. Generally, the sequences are the output of searching the query against the UniRef90 database [29]. In this study, the multiple sequence alignment of each analyzed protein (target sequence) was obtained by searching the UniRef90 database of non-redundant protein sequences using the default five search iterations of the profile HMM homology search tool jackhmmer. The alignments generated by the jackhmmer tool were directly processed by Sibe and converted into FASTA format, then the aligned sequences in each MSA were extracted and trimmed to remove poor quality sequences and improve efficiency in capturing the evolutionary information. In the trimming step, gaps were filtered according to both the column positions (amino acids in the query sequences) and rows (each protein sequence) in the MSA. As a result, the MSAs were post-processed to exclude sites of each sequence with more than 30% of gaps and to exclude sequences with less than 50% alignment to the target sequence. In our instructive example, we show in detail the process from sequence alignment to identifying positionally conserved couplings and applying those results to protein design and folding prediction.

Sibe incorporates statistical potentials derived from protein sequences (energy-like coevolution) and structure information (energetic potentials [28]) for protein design and folding, respectively. All of the calculations described in this work were carried out within the Sibe software suite and followed the same basic method. In computational protein design, within Sibe, large-scale protein sequences were generated by the dead-end elimination (DEE) algorithm [30] according to the statistical (energy-like) potentials inferred from MSA. Mutations occurred in a wild type protein sequence with the guidance of sequence energy-like potentials and were assessed by a metropolis criterion. Inferences of residue-contacts were also estimated from the MSA as a constraint to aid Sibe in protein folding and structure predictions. Combining such analysis with predicted constraints of torsion angles (*ϕ*, *ψ*) by a convolution neural network model [31], we performed iterative folding predictions using a Markov Chain Monte Carlo protocol [32] on a set of three representative proteins.

### Statistical analysis on sequences

In this study, the use of Sibe was focused on coevolution at the residue level including positionally conserved couplings and statistical potentials derived from the site bias of residues and the pairwise couplings of residues. We perform sequence statistics on a multiple sequence alignment and apply Sibe to capture the amino acid covariances and conservation for evolutionary inferences, then compute residue blocks. Given an MSA of *N* sequences by *L* positions, denoted as $\mathbf {M}=(M_{i}^{k})$, we can obtain an amino acid frequency at an individual position is $f_{i}(A)=\frac {1}{N}\sum _{k}\delta _{A,M_{i}^{k}}$, where *δ*=1 if sequence *k* has amino acid *A* at position *i*, otherwise *δ*=0. Similarly, a joint frequency of amino acid between a pair of positions is formulated as $f_{ij}(A,B)=\frac {1}{N}\sum _{k}\delta _{A,M_{i}^{k}}\delta _{B,M_{j}^{k}}$.

Here, the example of the human *β*_2_AR protein is used to show how Sibe can capture the couplings among residues and generate an energy-like potential derived from site bias of residues and pairwise couplings of residues. First, we compared the sequence of the target protein with the UniRef90 database [29] and obtained 221,306 sequences. Then we launched Sibe to analyze the MSA of the human *β*_2_AR protein sequences, and demonstrated how the statistical energetic potentials derived from the MSA can be used as evolutionary constraints for protein design.

To capture couplings between pairs of residues, we employ the Kullback-Leibler relative entropy [33] to measure how different the observed amino acid *A* at position *i* would be if *A* randomly occurred with an expected probability distribution [5]. The definition of the relative entropy is presented as follows,

1 $$  D_{i}(A)=f_{i}(A)\ln\left(\frac{f_{i}(A)}{p(A)}\right)+(1-f_{i}(A))\ln\left(\frac{1-f_{i}(A)}{1-p(A)}\right),  $$

where *p*(·) is the background probability.

To capture partial interactions, a global statistical model (DCA-based [6, 7, 9, 13]) is used to infer direct interaction information between pairwise residues. Here, we describe how to use Sibe to capture direct couplings from a given MSA using the model and create an energy-like potential for designing a variant of the human *β*_2_AR protein. Given the MSA, we can easily compute the single site frequency *f*_*i*_(*A*_*i*_) and joint frequency *f*_*ij*_(*A*_*i*_,*A*_*j*_). To maximize the entropy of the observed probabilities, we can calculate the effective pair couplings and single site bias to meet the maximal agreement between the distribution of expected frequencies and the probability model of actually observed frequencies.

2 $$ \left\{ \begin{array}{l} P_{i}(A_{i})=\sum\limits_{A_{k}|k=i}P(A_{1},A_{2},\cdots,A_{L})=f_{i}(A_{i})\\ P_{ij}(A_{i},A_{j})=\sum\limits_{A_{k}|k=i,j}P(A_{1},A_{2},\cdots,A_{L})=f_{ij}(A_{i},A_{j}) \end{array} \right.  $$

Maximizing the entropy of the probability model, we can get the following statistical model,

3 $$  P(A_{1},A_{2},\cdots,A_{L})=\frac{1}{Z}\exp\left\{\sum\limits_{i< j}e_{ij}(A_{i},A_{j})+\sum\limits_{i}h_{i}(A_{i})\right\}\!,  $$

where *Z* is a normalization constant, *e*_*ij*_(·,·) is a pairwise coupling, and *h*_*i*_(·) is a single site bias.

Evolutionary information based methods have been reported to be correlated with protein stability and can help design targeted single-point mutations [23, 34, 35]. For example, Best et al. [35] demonstrated that the DCA method can be used to build a sequence fitness landscape that can be an effective guide for protein rational design. As reported, most of the predicted high-fitness sequences of three proteins stably folded to the target structures in experiments. Ranganathan et al. [12] used coevolution between residues to create a stable and functional WW domain using the SCA approach by swapping pairwise coupled residues between sequences to maximize the similarity between designs and the natural alignment. However, it still remains challenging to completely disentangle direct and indirect couplings between residues, so they are not always reliable resources for guiding rational protein design. Here, we suggest that positionally conserved couplings between pairwise residues preserve large amounts of coevolutionary information resulting in higher reliability of rational protein design. Sibe provides an end-to-end platform for protein folding simulations and design in silico from protein primary sequence using conserved epistasis among amino acids. The main difference between our method and previous methods is that the conserved epistasis is estimated in order to capture potential amino acids that could contribute to function, since proteins evolve for function but not necessarily stability. In silico, Sibe allows us to detect mutations that may significantly guide protein engineering starting from a given sequence and then driving its folding by providing detailed pathway.

Site biases *h*_*i*_ and couplings *e*_*ij*_ can be estimated from the same MSA used for inferences of the positional conservation. Accordingly, positionally conserved couplings are computed from a combination of relative entropy from Eq. (1) with the site biases and pairwise couplings from Eq. (3) by using a sufficiently large and diverse MSA of a given target protein *τ*, as defined in the Eq. (4).

4 $$  E(\tau)=\sum\limits_{i< j}e_{ij}(\tau_{i}, \tau_{j})|(D_{i(j)}>\sigma)+\sum\limits_{i}h_{i}(\tau_{i})|(D_{i}>\sigma),  $$

where *σ* is a constant threshold for filtering amino acids at specific positions that are not conserved.

Rooting in Eq. (4), Sibe captures the positionally conserved couplings among residues from the MSA, which contribute to evolutionary constraints for both protein folding and design. In the following paragraphs, we demonstrate that statistically inferred information for basic evolutionary principles, such as positional conservations and coupled-evolution, can be used to predict protein structures and rationally design a diverse set of more efficient proteins.

## Results

**Fig. 1:**
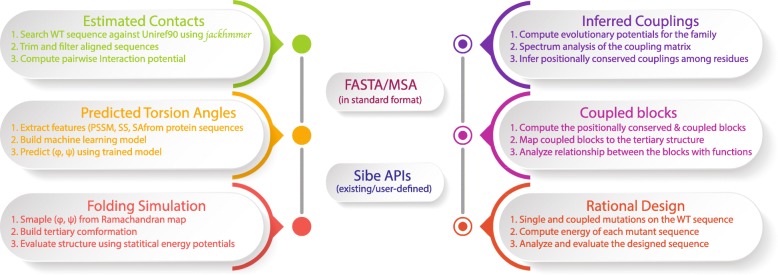
The flowchart of in silico protein folding and design in Sibe using evolutionary couplings as drivers

Written in C++, Sibe allows for the rapid analysis of long protein sequences and captures the evolution-based information for protein folding, design, and structure prediction (as illustrated in Fig. 1). In this section, we will describe how evolutionary coupling analysis in Sibe (as shown in Fig. 1) functions for a human *β*_2_-adrenergic receptor (*β*_2_AR) protein, which is a critical eukaryotic signal transduction protein that communicates across the lipid bilayer via recognition and binding of diffusible ligands for significant biological activities [27]. Understanding its sequence evolution can provide insight into its function and help in the design of better drugs and therapeutics. We use Sibe to study the human *β*_2_AR protein and demonstrate that Sibe can identify significant positionally conserved couplings and important structural features that have been linked to ligand binding activities.

### Protein sequence design

A great testing ground for the sequence to structure paradigm is protein design [36]. However, it is challenging to computationally assay for function in the large sequence space of an amino acid sequence [23] (e.g. a protein of 25 amino acids has a space of 20^25^). This challenge creates two major questions we approached using Sibe. First, how can we explore the large sequence space to capture key mutations that relate to the functional roles of a protein? Second, how can evolutionary information contained within amino acid sequences contribute to protein evolution (e.g. via establishing kinetic and thermodynamic stability [37]) and how can we use that information to design proteins with novel functions?

**Fig. 2:**
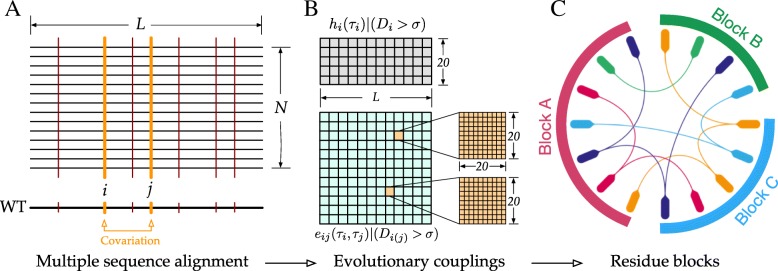
Analysis of evolutionary couplings. Starting from a given WT protein amino acid sequence, we apply Sibe to estimate the site bias of and pairwise couplings between amino acids as a statistical potential for protein design in silico. and a bias energy function (residue-contact) in protein folding. **a** Detection of covariations (e.g., at the *i*th and *j*th positions colored in orange) and conserved amino acids (e.g., positions colored in dark red) from the multiple sequence alignment of *N* sequences by *L* positions obtained by searching the WT sequence against the sequence database. **b** Analysis on the detected coupled and conserved amino acids results in an interaction potential. **c** residue blocks were achieved by independent component analysis and highlight each of them may have a distinct functional role in the protein family

To address these questions, we used Sibe to facilitate protein design and attempted to uncover the biophysical rules governing protein folding. In this section, we will use the human *β*_2_AR protein [27] to illustrate how Sibe functions for protein design in silico from sequence analysis. The Kullback-Leibler relative entropy of the human *β*_2_AR protein was computed from its MSA, as the information calculated from the relative entropy can remarkably reduce the potential complexity of the protein-design problem [12]. In Fig. 2 we provide an overview of the methodology for employing evolutionary couplings as a statistical energy-like potential (estimated from an MSA of a protein family using regularized maximum pseudolikelihood [38]) to constrain underlying protein sequence design (see also Additional file 1: Methods).

**Fig. 3:**
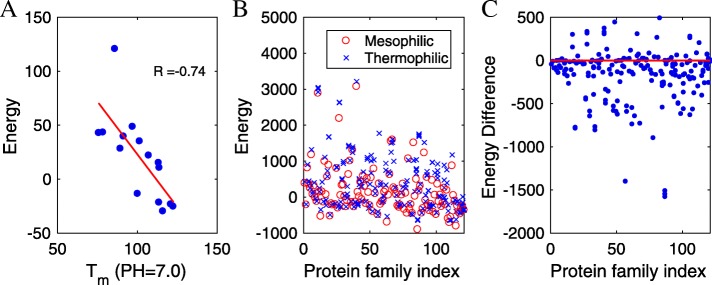
Comparisons between experimental and calculation results. **a** Correlation between the sequence energy and the transition temperatures for extant and ancestral Trx proteins at pH 7.0. **b** The sequence energies of the mesophilic and thermophilic proteins in the different protein families. **c** The energy difference calculated by Sibe between the mesophilic and thermophilic proteins in the same family

First, energy (as shown in Eq. 4) is significantly correlated with transition temperatures as measured by differential scanning calorimetry experiments for extant and ancestral Trx proteins [39], as shown in Fig. 3a. Although it does not suggest that protein function is related to temperature, it does indicate that computational inferences from multiple sequence alignment could make favorable contributions to rational design. We probed each given protein sequence from ref. [39] against the UniRef90 database [29] by HMMER [40] to prepare an MSA for the Trx protein family. The obtained MSA was used as an input to create site bias and coupling matrices. Accordingly, we calculated site bias and residues coupling energies for the sequences. To enhance the ability of the approach to distinguish proteins, we defined an energy equation *E*=*E*_*s*_+*α*·*E*_*c*_, where *E*_*s*_, *E*_*c*_ and *α* are site bias energy (contribution of a single amino acid to the whole sequence based on the statistical potential), coupling energy (contribution of pairwise amino acids), and a weight factor, respectively. According to stability analysis on the thioredoxin family (15 proteins) in ref. [39], we maximized the correlation between the total energies E and the transition temperatures by optimizing the weight factor (*α*). Based on the calculation, we got a maximum correlation factor approximately -0.74 when optimized *α* equals -0.43, as shown in Fig. 3a.

Further, to demonstrate the ability of the derived potential, we applied the derived potential value to distinguish mesophilic from thermophilic proteins within the same family (see Additional file 1: Table S1). We obtained MSAs of 136 different protein families that included mesophilic, thermophilic, moderately thermophilic, and extremely thermophilic proteins, then launched Sibe to infer the site bias and couplings matrices for test proteins from those 136 protein families. The calculated energy *E* was able to distinguish proteins from the same family for approximately 83.3% of the protein families tested. As illustrated in Fig. 3b, red circles and blue crosses indicate the energies of thermophilic and mesophilic proteins, respectively, calculated using potentials from Sibe. The blue circles in Fig. 3c show differences between the energies E of the mesophilic and thermophilic proteins within the same families. Thus, as demonstrated with these two experimental data sets, Sibe is able to distinguish mesophilic proteins from thermophilic proteins depending on sequence energy potential.

**Fig. 4:**
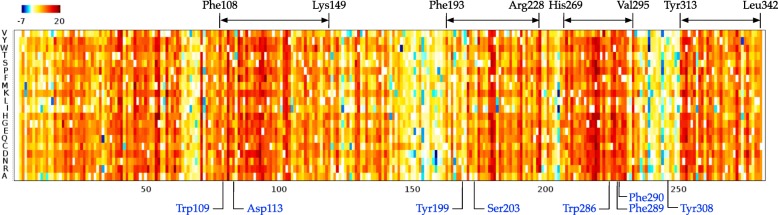
Effects of point mutations on the human *β*_2_AR protein. Substitutions at each position with negative *ΔE* values are predicted to be deleterious; while those that are positive are predicted to be tolerated. Neutral substitutions are marked in 0

We next investigated whether Sibe could be used in protein design using the evolutionary information inherent in an MSA. We conducted a computational design study of the human *β*_2_AR protein with the goal of capturing the coevolutionary information encoded in the natural evolutionary process in order to design a new stable *β*_2_AR variant that is likely to be functional (with in vitro validation). We generated an MSA using human *β*_2_AR homologous proteins consisting of 221,306 sequences (Additional file 1: Method), and the similarity between pairwise amino acid sequences was computed (illustrated in Additional file 1: Figure S1). We calculated the changes in energy for point mutations (Fig. 4), and found that potential mutants are rare (i.e. high energy) in the regions between residues Phe108-Lys149, Phe193-Arg228, His269-Val295, and Tyr313-Leu342 (marked on top of Fig. 4), as well as regions near the carazolol ligand binding sites consisting of residues Trp109, Asp113, Tyr199, Ser203, Trp286, Phe289, Phe290, and Tyr308 (colored in blue at the bottom of Fig. 4) [27].

**Fig. 5:**
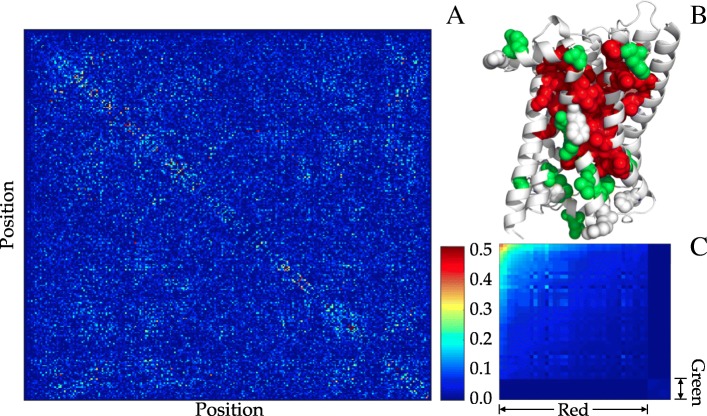
Matrix of pairwise residue-interactions for the human *β*_2_AR protein. **a** Evolutionary interactions inferred by PCC between pairwise residues. **b** Two interaction ‘blocks’ mapped to the tertiary structure. **c** Coupling matrix reordered to highlight two interaction blocks. Those blocks show that the residues in the same block may contribute similarly functions to the protein

We also analyzed a matrix consisting of coupling terms *e*_*ij*_ in Eq. (3) between pairs of amino acids by independent component (IC) analysis [41]. According to IC analysis, just the top two eigenvalues are statistically significant. These two top eigenmodes of the coupling matrix (as illustrated in Fig. 5a that shows all the coupled interactions between pairwise residues) are transformed into two independent components that define independent blocks, which are groups of amino acids that are physically connected in the tertiary structure and may be functionally correlated. Figure 5b and c show that Sibe successfully identified two blocks for human *β*_2_AR protein, one of which consists of 39 residues covering functional sites of the protein [27] (red block, Fig. 5). These results suggest that Sibe can detect coupled evolution among residues important for protein function and may contribute to designing stabilized proteins by suggesting residues for mutation based on coevolutionary information.

**Fig. 6:**
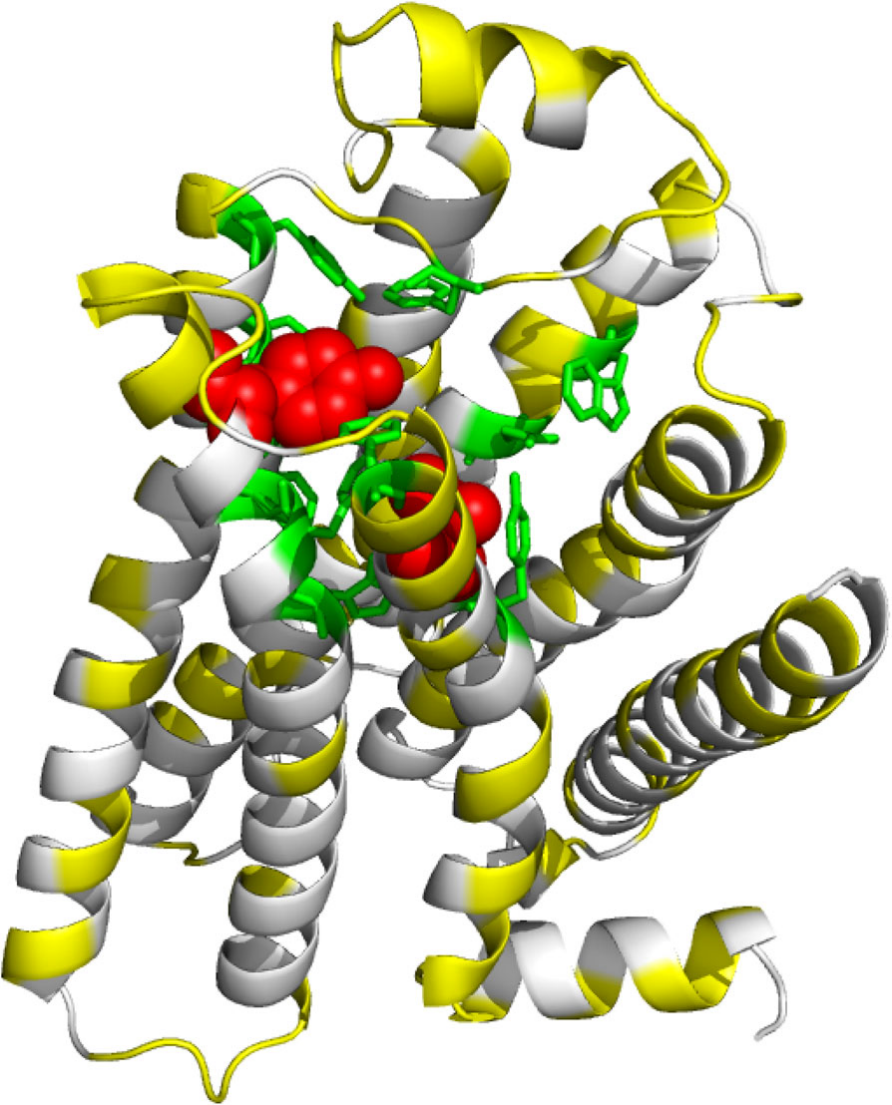
Sibe computationally designed a mutant sequence of the human *β*_2_AR protein (PDB ID: 2RH1) [27] that is mapped to it tertiary structure. Yellow, green and red indicate mutations (made by Sibe) occurred at common sites, ligand-binding sites, and experimentally mutated functional sites, respectively

Sibe can also be used to identify the designed protein sequence with the lowest energy. The critical features of this protein design protocol are described in Additional file 1: Figure S2 and the full method is described in the supplementary materials. To assess whether this approach can produce a more stable protein from coupling constraints encoded in the MSA according to coevolution-derived energies, we performed the DEE minimization protocol [30] on protein sequence design. For each starting sequence, five thousand independent simulations, each consisting of a trial of sequence design with maximum iterations of 100,000, were performed to obtain the lowest energy from 500 trials (shown in sequence logo in Additional file 1: Figure S3). In Fig. 6, we show one such analyzed protein sequence, the ligand-binding site in human *β*_2_AR protein, shown in green. Within this site, extensive interactions occur between the *β*_2_AR protein and carazolol at Phe289, Phe290 and Trp286 [27]. In the computationally designed human *β*_2_AR protein, we obtained three ligand-binding site mutants [27], which may have altered function compared to wild type protein upon experimental demonstration.

### Protein folding and structure prediction

The prediction of protein structures has been a long-standing challenge and numerous advances have been made towards determination of the three-dimensional structure of a protein from its amino acid sequence [28, 42–44]. However, there are remaining challenges regarding efficient computational methods for interpretation of large sequencing data for protein families and the development of rapid structure modeling approaches. Addressing this gap is especially important as recently, due to efforts in metagenome sequence projects, the number of protein sequences is growing considerably faster than ever before [25, 45]. The gap between a protein sequence and its unknown structure can be largely reduced by taking advantage of progress in statistical analysis of both protein sequences and known structures. Moreover, known coevolution among amino acids enhances the capacities of existing computational approaches in predicting contacts between protein residues [6, 7, 9], and such predicted constraints can provide an accurate way to model a protein of unknown structure [25].

**Fig. 7:**
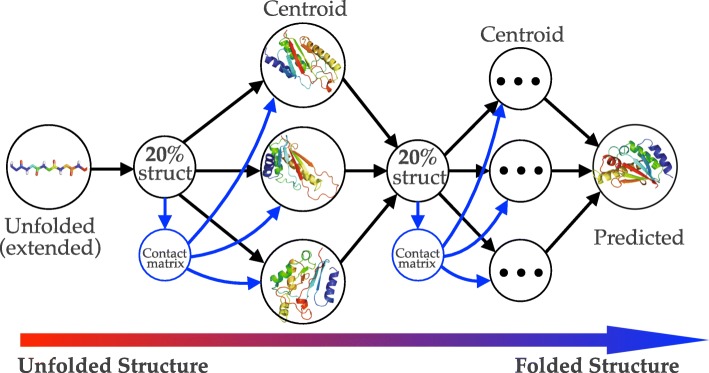
Iterative structure prediction guided by coevolution. Starting from unfolded (extended) structure, Sibe incorporates residue-contacts derived from coupling analysis on the MSA and averaged residue-contacts from predicted structures (previous round) with lower energy (best 20%) as constraints to iteratively predict the tertiary structures of targets

To assess structure prediction by Sibe, we carried out calculations on an instructive example consisting of three representative proteins following the steps shown on the left side of Fig. 1. The three proteins chosen were the low molecular weight protein tyrosine phosphatase YwlE (PDB ID: 1ZGG), a flagellar capping protein (PDB ID: 5FHY), and the E. coli MCE protein MlaD (PDB ID: 5UW2). Positionally conserved couplings and predicted protein torsion angle (*ϕ*, *ψ*) constraints for each protein were used for analysis [46]. We present an iterative framework (Fig. 7) to fold a protein using statistical potentials [28] and coevolution constraints derived from its sequence alignment (as described above). The iterative prediction uses a Markov Chain Monte Carlo protocol (Additional file 1: Figure S4 and Method) and includes multiple rounds in which the predicted constraints (e.g. torsion angles, residue-contacts) can be collected from the previous round to guide and bias the prediction.

As each potential confirmation of a protein is drawn from the same Ramachandran map distribution according to the given amino acid sequence, the conformations generated by the Markov Chain Monte Carlo method are partially correlated with each other. In each folding simulation, starting from a query sequence, we generated five hundred initial conformations to trigger structure predictions by iteratively biasing the prediction from constraints. For example, Sibe was used to iteratively predict the tertiary structure of the YwlE protein using the constraints of residue-contacts inferred from its MSA and predicted torsion angles (which are used to increase probabilities of on the Ramachandran map distribution) predicted by *Phsior* [46]. After 500 simulations were converged, we chose the one hundred predicted structures with the lowest energy (20% of all structures) and calculated the averaged residue-contacts (for spatial interactions among residues) and torsion angles (defined as the square ranges for each pair of *ϕ* and *ψ* located in $\left [\phi _{i}-\phi ^{pred}_{i}\right ]^{2}+\left [\psi -\psi ^{pred}_{i}\right ]^{2}=[25^{\circ }]^{2}$) (see also Additional file 1: Methods).

**Fig. 8:**
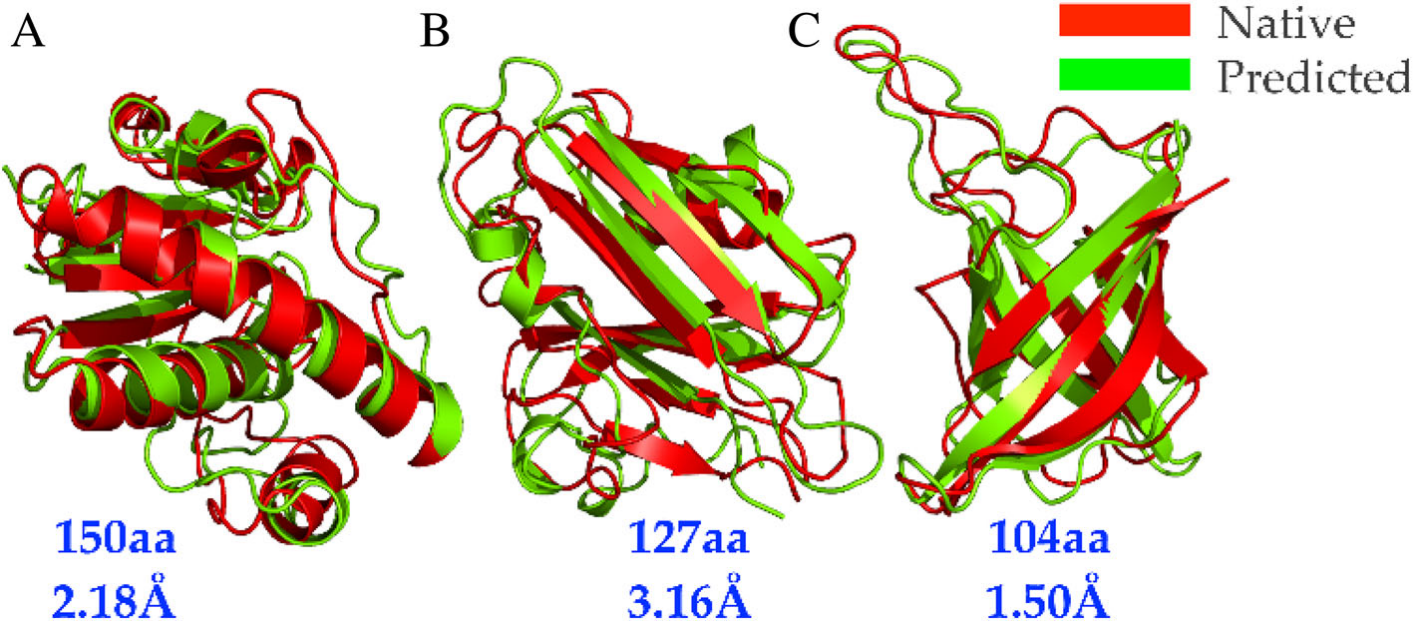
Comparison of models predicted by Sibe (green) to the crystal structures (red). The models accurately recapitulate the structural details of the named proteins. The root-mean-square deviation (RMSD) of each protein was computed by PyMOL software [47] as shown, and the TM-scores are as follows: **a** YwlE (PDB ID: 1ZGG, RMSD 2.18Å, TM-score 0.76), **b** the flagellar capping protein (PDB ID: 5FHY, RMSD 3.16Å, TM-score 0.64), **c** the E. coli MCE protein MlaD (free modeling target in CASP12, PDB ID: 5UW2, RMSD 1.50Å, TM-score 0.80)

We then compared the predicted models for the target proteins in comparison to the crystal structures (shown in Fig. 8) and we compared the predictions of Sibe to those of the EVfold-server [9] (Additional file 1: Table S2 and Figure S5). The predicted results of protein tertiary structures are close to the actual crystal structures and thus show the capability of Sibe’s protein structure prediction module. As a de novo predictor, Sibe has better performance in folding proteins of more than 100 amino acids in CPU hours (as illustrated in Additional file 1: Figure S6).

## Discussion

Since the introduction of statistical analyses of proteins to the biophysics community, improvements of algorithms for inferring couplings between pairwise residues has been the focus of intense study. Although the practical implementation of these algorithms has produced several historic packages that were strongly tied to the best practices in basic research on protein sequence analysis and folding, software rewrites have been common due to the fast-moving pace of experimental research.

The success of such statistical software depends in part on the method for deriving positionally conserved couplings to detect amino acid variations, and the easy interface modules presented in Sibe lay the groundwork for drawing interpretable conclusions from protein sequence data about its folding for and design studies in silico. Due to rapid advances in the software suite Sibe, a variety of functional modules are available to researchers for analyzing protein sequences, protein folding, and design in silico. In the second example presented in this paper, we demonstrate how Sibe’s implementation of an iteratively biasing conformation search can be used to predict the tertiary structures of proteins from their amino acid sequences based on statistical potentials of protein sequences and structures. Due to the limited diversity in the MSA of a given protein family, Sibe is imperfect in capturing significant co-variants as coevolutionary constraints for protein design and structure prediction. Accordingly, the remaining challenges are how to enrich the diversity of information in the MSAs and how to efficiently detect important coevolutionary couplings between pairwise amino acids and distinguish those couplings from biases that arise within protein families of lower diversity. Future work may focus on addressing those issues by the extension and improvement of Sibe.

## Conclusions

We report here that a software suite (Sibe) provides an analytical and computational tool for protein sequence data analysis, in silico protein folding and design. All modules in Sibe are implemented in the C++ programming language, and Sibe employs extensible application programming interfaces (APIs) in both C++ and/or Python, which allows rapid analysis of large amounts of protein sequence data for boosting abilities in protein design and folding predictions. Through two instructive applications, we have demonstrated the capabilities of Sibe in extracting meaningful information hidden behind ’big data’ to infer coevolutionary information encoded in amino acid sequences of proteins. In the first example, we applied Sibe to analyze protein sequences for studying the relationships between protein sequence and thermostability, with potential applications in rational design of proteins. We highlight the statistical potential of positionally conserved couplings (PCCs) among residues that are accelerated by graphics processing unit (GPU) computing. In the second application, we demonstrate how Sibe can simulate protein folding using PCCs as a driver and biases that are predicted by machine learning. We account for the overwhelming nature of simulating protein folding by iteratively fixing the residue-contacts and constraints of torsion angles.

Generally, Sibe’s power and simple architecture are dependent on expressive and functional modules, which focus on extending methods specifically designed for scientific applications. Understanding the coevolutionary process from metagenome sequence data provides thermodynamic insights into a protein’s evolution, which can aid in the design of more efficient proteins. We hope that the methodology of protein design will have future applications in chemistry, bioremediation, drug design, and drug discovery.

## Additional file

Additional file 1 Supplementary material. (PDF 1535 kb)

## Data Availability

The Sibe software suite can be obtained at request for non-commercial use and its installation documentation is available on its web-server at: http://wyu.dgist.ac.kr/sibe.

## Abbreviations

*β*_2_AR: human *β*-adrenergic receptor
_2_API: Application programming interface
DCA: Dirct coupling analysis
DEE: dead-end elimination
GPCR: G-protein-coupled receptor
GPU: Graphics processing unit
MSA: Multiple sequence alignment
PCC: Positionally conserved coupling
SCA: Statistical coupling analysis
