# Supplementary material for: Sibe: a computation tool to apply protein sequence statistics to predict folding and design in silico
Source: BMC Bioinformatics. 2019 Sep 6;20:455. doi: 10.1186/s12859-019-2984-1 (PMC6728967; doi:10.1186/s12859-019-2984-1)
Supplement: Supplementary file 1 — Supplementary material. (PDF 1535 kb) [file 12859_2019_2984_MOESM1_ESM.pdf]

Supplemental Material
Sibe: a computation tool to apply protein sequence statistics to predict folding and design *in silico*

N. J. Cheung, W. Yu

Methods and materials

Sibe is a software suite rooting in statistical models, optimization solvers and energy functions for both protein studies and data mining. In this study, within Sibe statistical information inferred from multiple sequence alignment is generated and applied to protein folding, structure prediction and design. Two main tasks were presented by two instructive examples, in which we are to show the ability of Sibe in folding and designing a protein.

Analysis on protein sequences

First of all, we search a query sequence against the UniRef90 database [1] by HMMER [2] and then prepare its sequence alignment. The aligned sequence can be directly used to infer evolutionary information, since the HMMER tool provides the best ContTest score [3] and comparable score in the QuanTest [4]. The following command lines are used to collect the aligned sequences,

```
jackhmmer \
  -N 5 \
 --notextw \
  -A out.sto \
 --tblout out_tbl.out \
 --domtblout out_domtbl.out \
  -E 0.01 \
 --popen 0.25 \
 --pextend 0.4 \
   query_fasta \
   uniref90.fasta
```

We prepared a wild type sequence of the human β_2AR protein as the example for protein sequence analysis. By running `jackhmmer`, we got a raw sequence alignment and then trimmed them by a command line as follows,

```
sibe sequence_trim \
 -msa=raw_sequence_alignment.msa
```

The command line is to trim the aligned sequences according to the first sequence (query sequence) in the input sequence alignment.

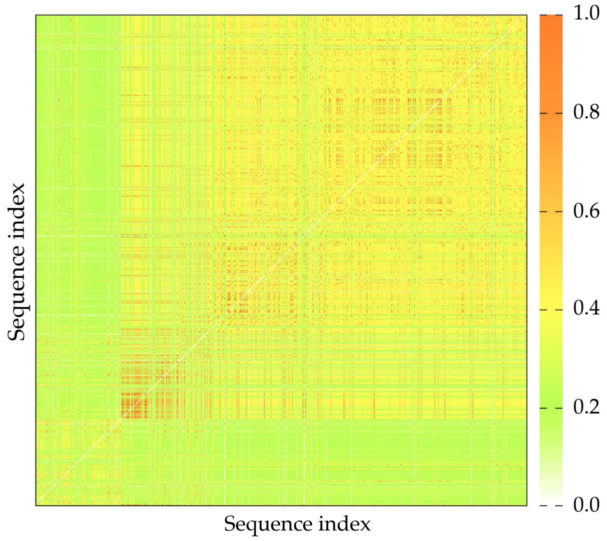

Figure S1: Similarity between pairs of sequences.

At the begin of sequence analysis, we use command `sequence_stats` in Sibe to conduct a naive examination of the sequence space described by the alignment. Sibe will also present a Shell script for plotting figures of the output. By running the script, the computed a matrix of similarity between pairs of sequences can be plotted as illustrated in Fig. S5. The matrix representation gives the fraction of amino acids that are common between the pairwise sequences. As another computational results, the bar representation of positional conservation is shown in Fig. 3.

```
sibe sequence_stats \
-msa=raw_sequence_alignment.msa
```

Computational protein design

Validations of the calculations

In computational protein design, we first demonstrate that the estimated sequence potential from MSA can distinguish the mesophilic and thermophilic proteins. Likewise, we prepared a MSA for each family and then inferred the energy-like potential from the MSA by running command line as follows,

```
sibe sequence_coupling \
 -msa=sequence_alignment_trimed.aln
```

Based the obtained potential, we calculated the energies of the sequences using the same estimated potential if the sequences are in the same family. The command line is to compute the sequence energy as follows,

```
sibe sequence_energy \
 -msa=the_sequences_require_energy_calculation.aln \
 -mat=sequence_potential.mat
```

The calculations are shown in Fig. 6. We demonstrated that the computational results have high correlation (Pearson correlation coefficient ~ -0.74) with the experimental data [5], and the

estimated potential and the model were validated to be efficient in distinguishing the mesophilic from thermophilic proteins, 24 of all protein families as listed in Table S1, others can be find in ref. [6].

Table S1: Meso- & thermo-philic proteins used in this study

No.	Family name	PDB ID	Source organism (meso-/thermo-philic)
1	Transcription initation	1volA	Human (meso)
	factor IIb	1aisB	Pyrococcus woesei (thermo/100°C)
2	Superoxide dismutase	1ar4A	Propionibacterium freudenreichii (meso)
	(Mn- or Fe-dependent)	3mdsA	Thermus thermophilus (thermo/75°C)
3	Glutamate dehydrogenase	1hrdA	Clostridium symbiosum (meso)
		1gtmA	Pyrococcus furiosus (thermo/100°C)
4	Malate dehydrogenase	4mdhA	Pig heart (meso)
		1bmdA	Thermus flavus (thermo/72.5°C)
5	Phycocyanin alpha chain	1cpcA	Fremyella diplosiphon (meso)
		1liaA	Polysiphonia urceolata (meso)
		1phnA	Cyanidium caldarium (thermo/45°C)
6	Signal recognition particle	1fts	Escherichia coli (meso)
	(receptor)	1ffh	Thermus aquaticus (thermo/72.5°C)
7	Ferredoxin	1fxd	Desulfovibrio gigas (meso)
		1fxrA	Desulfovibrio africanus (meso)
		1vjw	Thermotoga maritima (thermo/80°C)
8	Subtilisin	2pkc	Tritirachium album limber (meso)
		1thm	Thermoactinomyces vulgaris, 60°C)
9	Neutral protease	1npc	Bacillus cereus (meso)
	(thermolysin)	1lnfE	Bacillus thermoproteolyticus (thermo/52.5°C)
10	Rubredoxin	6rxn	Desulfovibrio desulfuricans (meso)
		1caa	Pyrococcus furiosus (thermo/100°C)
11	Cyclodextrin	1cdg	Bacillus circulans strain 251 (meso)
	glycosyltransferase	1cgt	Bacillus circulans strain 8 (meso)
		1pamA	Bacillus sp. 1011 (meso)
		1ciu	Thermoanaerobacterium thermosulfurigenes (thermo/60°C)
		1cyg	Bacillus stearothermophilus (thermo/52.5°C)
12	Phycocyanin beta chain	1cpcB	Fremyella diplosiphon (meso)
		1liaB	Polysiphonia urceolata (meso)
		1phnB	Cyanidium caldarium (thermo/45°C)
13	3-Phosphoglycerate kinase	1qpg	Yeast (meso)
		1vpe	Thermotoga maritima (thermo/80°C)
14	Glyceraldehyde-3-phosphate	1a7kA	Leishmania mexicana (meso)
	dehydrogenase	1hdgO	Thermotoga maritima (thermo/80°C)
15	Xylanase (I)	1ukrA	Aspergillus niger (meso)
		1yna	Thermomyces lanuginosus (thermo/45°C)
16	Xylanase (II)	1clxA	Pseudomonas fluorescens (meso)
		1xyzA	Clostridium thermocellum (thermo/60°C)
17	TATA box binding protein	1cdwA	Human (meso)
		1vokA	Arabidopsis thaliana (meso)
		1pczA	Pyrococcus woesei (thermo/100°C)

18	Adenylate kinase	2ak3A	Bovine (meso)
		1ukz	Yeast (meso)
		1zip	Bacillus stearothermophilus (thermo/52.5°C)
19	Carboxypeptidase	1nsa	Pig (meso)
		1pca	Pig (meso)
		1obr	Thermoactinomyces vulgaris (thermo/55°C)
20	Ornithine carbamoyltransferase	2otcA	Escherichia coli (meso)
		1a1s	Pyrococcus furiosus (thermo/100°C)
21	Pyrophosphatase	1obwA	Escherichia coli (meso)
		2prd	Thermus thermophilus (thermo/72.5°C)
22	CheY protein	3chy	Escherichia coli (meso)
		2chf	Salmonella typhimurium (meso)
		1tmy	Thermotoga maritima (thermo/80°C)
23	Phosphofructokinase	1pfkA	Escherichia coli (meso)
		4pfk	Bacillus stearothermophilus (thermo/52.5°C)
24	Triacylglycerol acylhydrolase	1lgyA	Rhizopus niveus (meso)
		1tib	Humicola lanuginosa (thermo/50°C)
		3tgl	Rhizomucor miehei (thermo/45°C

The effects of point mutations were computed from the potential by running following command line. The matrix representation of the computational results is shown in Fig. 7.

```
sibe point_mutation \
 -fastx=query_fasta_WT.fasta \
 -mat=input_sequence_potential.mat
```

Protein design protocol

Using the algorithm described by Desmet et al. [7], the DEE procedure for each trajectory starts with a given wild type sequence. The sequence converges to a local energy minimum by gradually decreasing the temperature. As illustrated in Fig. S2, starting from a given wild type sequence (`WT_sequence`), we launched a DEE algorithm to optimize the mutant sequence base on the energy-like potential (`sequence _potential`) inferred from the MSA. According to the Metropolis criterion, the design protocol will accept or reject a new mutant that may occur in the wild type sequence, and the change is accepted with probability,

$$P = \min\{r_p, e^{-\Delta E/t}\} \tag{1}$$

where $r_p = 1$, and ΔE is the energy difference between the new and old designed sequences.

By running the following command line, we can get a trajectory (`the_designed_sequence .trajct`) of mutant sequences starting from the wild type sequence.

```
sibe sequence_design \
 -fastx=WT_sequence \
 -mat=sequence_potential.mat \
 -dseq=the_designed_sequence.trajct \
 -iterations=100000
```

From the design protocol, we got five hundreds trajectories of the designed sequences, and the sequence with lowest energy in each trajectory was collected for visualization as shown in Fig. S4.

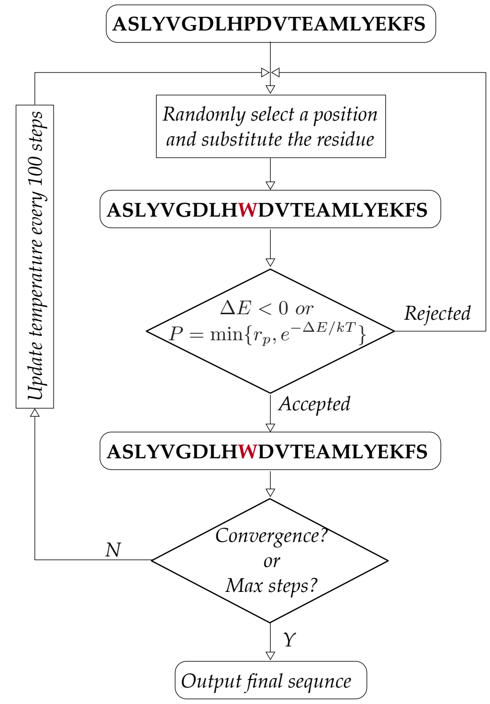

Figure S2: Flowchart of the computational protein design.

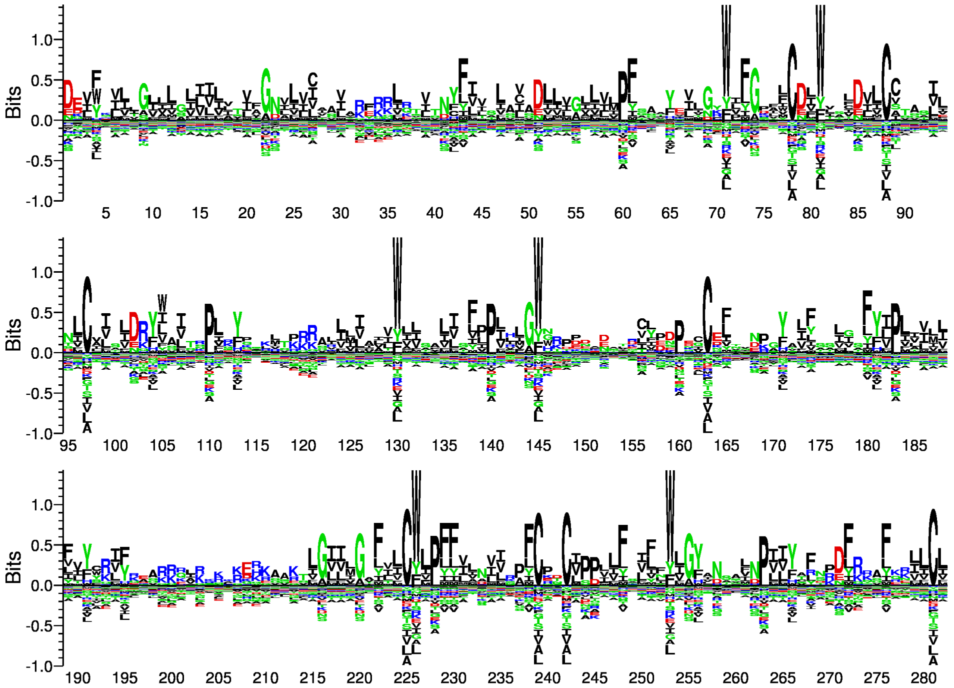

Figure S3: Visualization of the computationally designed sequences with the lowest energy in each trajectory.

Protein folding and structure prediction

In Sibe, MCMC algorithm is used for protein conformation sampling from individual (ϕ, ψ) distributions (Ramachandran maps), while all other angles and bond lengths are fixed at their ideal

values. A single round involves 500 individual MCMC folding simulations that are run using specialized (ϕ, ψ) backbone sampling procedures and the energy functions (as described in ref. [8]) in a protein representation containing the backbone and C_β atoms. Within the simulation, MCMC provides a general solution to protein folding and structure prediction prevalent in scientific research. As described in ref. [8], Sibe utilizes the same moving sets and energy functions. Additionally, Sibe employed predicted angles (ϕ, ψ) to increase the sampling probabilities in the Ramachandran map distribution, which also efficiently enhance the ability of the MCMC method during the simulation. The passing of constraints of torsion angles (ϕ, ψ) and residue-contacts from one round to another is repeated until convergence as illustrated in Fig. S4.

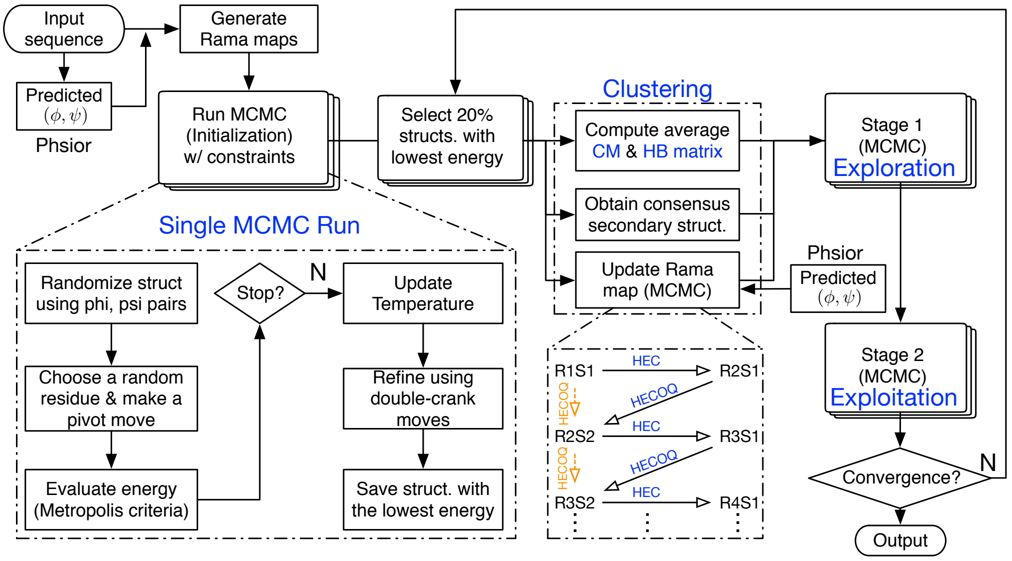

Figure S4: Flowchart of a single round in the iterative protein folding.

Constraints-guided sampling

In Sibe, we used the *Phsior* [9] to predict torsion angles (ϕ, ψ), which were applied to shift the Ramachandran map distribution and increase the probabilities p of (ϕ, ψ) of the ith residue located in circle $[\phi_i - \phi_i^{pred}]^2 + [\psi - \psi_i^{pred}]^2 = [25°]^2$ by increment of $0.75 * p$. In each round, a consensus secondary structure is obtained at every amino acid position, which is used to alter the Ramachandran map distribution. Combining with the predicted torsion angles (ϕ, ψ), we can make changes in the probabilities in each map of amino acids. This strategy leads to efficient sampling in the MCMC algorithm and accelerate the folding of a protein structure.

The residue-contacts (estimated by evolutionary couplings [10, 11]) are used as constraints in iterative simulations as illustrated in Fig. 10. The top L/3, L/2, L, 3L/2 (L is the length of a protein sequence) predicted contacts are used for 500 simulations in initial stage. Every 125 simulations utilize the same initial constraints of residue-contacts, e.g. top L/3 residue-contacts are used in the first 125 simulations while top L/2 residue-contacts are employed by another 125 simulations. This hierarchical application of constraints from residue-contacts is to help the Markov chain sample efficiently in conformation space.

Metropolis-Hastings sampling

In Sibe, we use a Markov chain to sample from Ramachandran maps distribution (π) (derived from high resolution PDB structures [8]). Accordingly, it is necessary to develop a transition for

the Markov chain, aiming to match the chains stationary distribution with the individual (ϕ, ψ) distributions. As an effective strategy to sample angular space (ϕ, ψ), a random-walk Markov chain uses the current state of a chain of conformations to propose a new state. Within Sibe, a Gaussian function centered on the current state $g(s|s^{(t-1)}) \sim \mathcal{N}(s^{(t-1)}, 1)$ is defined as the proposal function. This allows the algorithms to exploit the conformation space of the posterior–if a new conformation is similar to the last draw, then it is likely to be accepted. The proposal distribution $g(s|s^{(t-1)})$ is dependent only on the previous state in the Markov chain.

Starting from some random initial state $s^{(0)} \sim \pi^{(0)}$, the protocol first draws a potential state s from a proposal distribution $g(s|s^{(t-1)})$. Accordingly, in Sibe the Metropolis-Hastings algorithm is launched as the simplest form of random-walk MCMC protocol, which accepts or rejects a transition from protein conformation $s^{(t-1)}$ to s with probability α. The detailed criteria for accepting or rejecting a proposed state are defined as follows:

1. If $R(s) \geq R(s^{(t-1)})$, the proposed state s will be set as the next state in the Markov chain.

2. If $R(s) < R(s^{(t-1)})$, then the proposed state may still be accepted, but only randomly, and with a probability $\frac{R(s)}{R(s^{(t-1)})}$. The acceptance probability for the proposed state is calculated as follows,

$$P(s^{(t-1)} \to s) = \min\left(1, \frac{R(s)g(s \to s^{(t-1)})}{R(s)g(s^{(t-1)} \to s)}\right), \tag{2}$$

   where R is the density of the individual ϕ, ψ distribution of an amino acid, and g is the density of the proposal function for a transition from $s^{(t-1)}$ to s.

   The proposed state s is accepted if a random uniform number u is less than or equal to α. Otherwise, it will be rejected, and the current state will be set as the next state in the Markov chain.

Comparisons

We have compared our predictions to those of EVfold [10] on the three proteins. As shown in Table S2. All the results are computed from EVfold web-server, and the comparisons between EVfold and Sibe are reported in Table S2 in the supplementary material.

Table S2: Comparison of predictions between EVfold and Sibe.

	EVfold			Sibe
	RMSD (TMscore)			
	b†0.2	b†0.3	b†0.4	RMSD (TMscore)
1ZGG	3.51Å (0.71)	3.66Å (0.68)	4.51Å (0.61)	2.18Å (0.76)
5FHY	5.23Å (0.49)	-‡	3.75Å (0.61)	3.16Å (0.64)
5UW2	4.25Å (0.57)	4.53Å (0.54)	4.51Å (0.52)	1.50Å (0.80)

†: the multiple sequences were aligned at a bitscore.

‡: no results.

As shown in Fig. S5, the results were computed by the EVfold web-server at https://evcouplings.org. All the predictions are based on the default configurations on the server and the visualizations of the tertiary structures are presented by PyMol [12] for comparison to our results as shown in Table S2. As a *de novo* method, folding module in Sibe has better performance than that of EVfold, since Sibe provides folding details in the predictions and iteratively bias the folding.

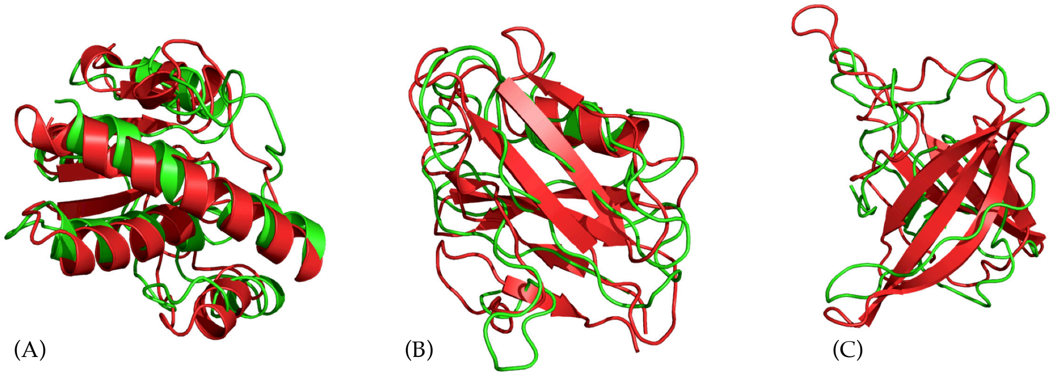

Figure S5: The comparison of models predicted by EVfold [10] (green) to the crystal structures (red). Top one Predicted structure of (A) the YwlE protein (1ZGG, bitscore = 0.2), (B) the flagellar capping (5FHY, bitscore = 0.4), and (C) the E. coli MCE protein MlaD (5UW2, bitscore = 0.2).

Running time

As shown in Fig. S6, the running time of different tasks (MSA, statistical potential, folding) are presented. For all the proteins used in the study, the *jackhmmer* tool took similar time to search the UniRef90 database for obtaining the MSA of each protein. The running time of both estimated potential and folding is highly dependent on the protein length and the number of sequences in the MSAs. For example, it took about 13 hours to infer the statistical potential for the human β_2AR protein due to its longer sequences and large number of sequences in its MSA as illustrated in Fig. S6(a). On distributed computational clusters, we conducted folding simulations on eighteen proteins [13] and computed the running time as shown in Fig. S6(b).

As illustrated in Fig. 1, it took much longer time to folding a protein into its tertiary structure comparing to that of calculating the potential.

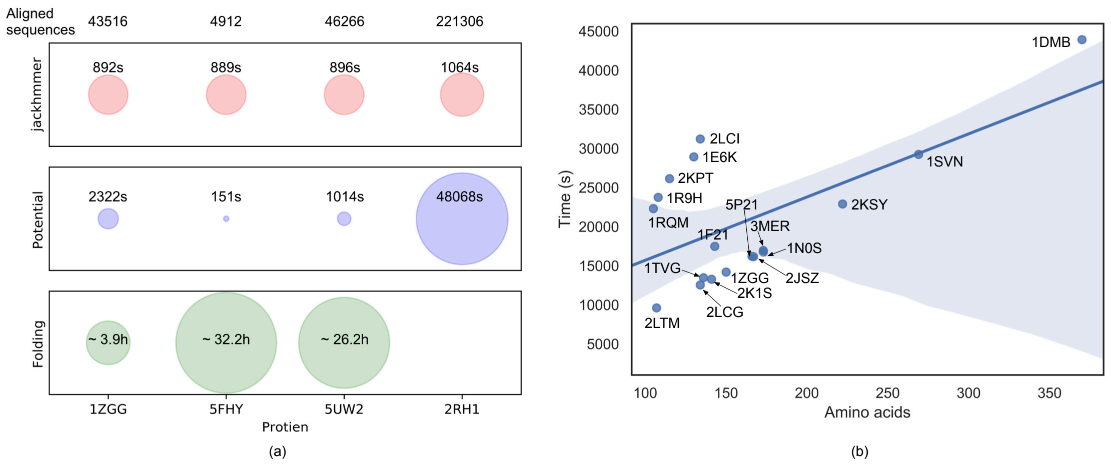

Figure S6: Running time of obtaining MSA, estimating sequence potential, and folding simulation.

References

[1] Baris E Suzek, Yuqi Wang, Hongzhan Huang, Peter B McGarvey, Cathy H Wu, UniProt Consortium, et al. Uniref clusters: a comprehensive and scalable alternative for improving sequence similarity searches. *Bioinformatics*, page btu739, 2014.

[2] Sean R. Eddy. Accelerated profile HMM searches. *PLOS Computational Biology*, 7(10):1–16, 10 2011.

[3] Gearóid Fox, Fabian Sievers, and Desmond G Higgins. Using de novo protein structure predictions to measure the quality of very large multiple sequence alignments. *Bioinformatics*, 32(6):814–820, 2015.

[4] Quan Le, Fabian Sievers, and Desmond G Higgins. Protein multiple sequence alignment benchmarking through secondary structure prediction. *Bioinformatics*, 33(9):1331–1337, 2017.

[5] Franco O. Tzul, Daniel Vasilchuk, and George I. Makhatadze. Evidence for the principle of minimal frustration in the evolution of protein folding landscapes. *Proceedings of the National Academy of Sciences*, 114(9):E1627–E1632, 2017.

[6] Todd J Taylor and Iosif I Vaisman. Discrimination of thermophilic and mesophilic proteins. *BMC structural biology*, 10(1):S5, 2010.

[7] John Desmet, Marc De Maeyer, and Ignace Lasters. The dead-end elimination theorem and its use in protein side-chain positioning. *Nature*, 356(6369):539, 1992.

[8] Aashish N Adhikari, Karl F Freed, and Tobin R Sosnick. *De novo* prediction of protein folding pathways and structure using the principle of sequential stabilization. *Proceedings of the National Academy of Sciences*, 109(43):17442–17447, 2012.

[9] Ngaam J. Cheung, W. Yu, J. M. Jumper, K. F. Freed, and T. R. Sosnick. *De novo* protein structure prediction using ultra fast molecular dynamics simulation. 2018.

[10] Debora S. Marks, Lucy J. Colwell, Robert Sheridan, Thomas A. Hopf, Andrea Pagnani, Riccardo Zecchina, and Chris Sander. Protein 3D structure computed from evolutionary sequence variation. *PLOS ONE*, 6(12):1–20, 12 2011.

[11] Debora S Marks, Thomas A Hopf, and Chris Sander. Protein structure prediction from sequence variation. *Nature Biotechnology*, 30:10721080, 2012.

[12] WL DeLano. The PyMOL molecular graphics system, version 1.2 r3pre, schrödinger, LLC. 2002.

[13] Ngaam J Cheung and Wookyung Yu. De novo protein structure prediction using ultra-fast molecular dynamics simulation. *PloS one*, 13(11):e0205819, 2018.
